# Towards graphane field emitters

**DOI:** 10.1039/c5ra20771a

**Published:** 2015-09-23

**Authors:** Shuyi Ding, Matthew T. Cole, Chi Li, Yanhuai Zhou, Clare M. Collins, Moon H. Kang, Richard J. Parmee, Wei Lei, Xiaobing Zhang, Qing Dai, William I. Milne, Baoping Wang

**Affiliations:** a Display R&D Center , School of Science and Engineering , Southeast University , Nanjing 210096 , P. R. China . Email: lw@seu.edu.cn; b Electrical Engineering Division , Department of Engineering , Cambridge University , Cambridge , CB3 0FA , UK . Email: mtc35@cam.ac.uk; c National Centre for Nanoscience & Technology , Beijing , 100190 , P. R. China . Email: daiq@nanoctr.cn; d School of Information Engineering , Nanjing Normal University Taizhou Collage , Taizhou , 225300 , P. R. China; e Quantum Nanoelectronics Research Center , Tokyo Institute of Technology , Tokyo , Japan

## Abstract

We report on the improved field emission performance of graphene foam (GF) following transient exposure to hydrogen plasma. The enhanced field emission mechanism associated with hydrogenation has been investigated using Fourier transform infrared spectroscopy, plasma spectrophotometry, Raman spectroscopy, and scanning electron microscopy. The observed enhanced electron emissionhas been attributed to an increase in the areal density of lattice defects and the formation of a partially hydrogenated, graphane-like material. The treated GF emitter demonstrated a much reduced macroscopic turn-on field (2.5 V μm^–1^), with an increased maximum current density from 0.21 mA cm^–2^ (pristine) to 8.27 mA cm^–2^ (treated). The treated GFs vertically orientated protrusions, after plasma etching, effectively increased the local electric field resulting in a 2.2-fold reduction in the turn-on electric field. The observed enhancement is further attributed to hydrogenation and the subsequent formation of a partially hydrogenated structured 2D material, which advantageously shifts the emitter work function. Alongside augmentation of the nominal crystallite size of the graphitic superstructure, surface bound species are believed to play a key role in the enhanced emission. The hydrogen plasma treatment was also noted to increase the emission spatial uniformity, with an approximate four times reduction in the per unit area variation in emission current density. Our findings suggest that plasma treatments, and particularly hydrogen and hydrogen-containing precursors, may provide an efficient, simple, and low cost means of realizing enhanced nanocarbon-based field emission devices *via* the engineered degradation of the nascent lattice, and adjustment of the surface work function.

## Abstract 2

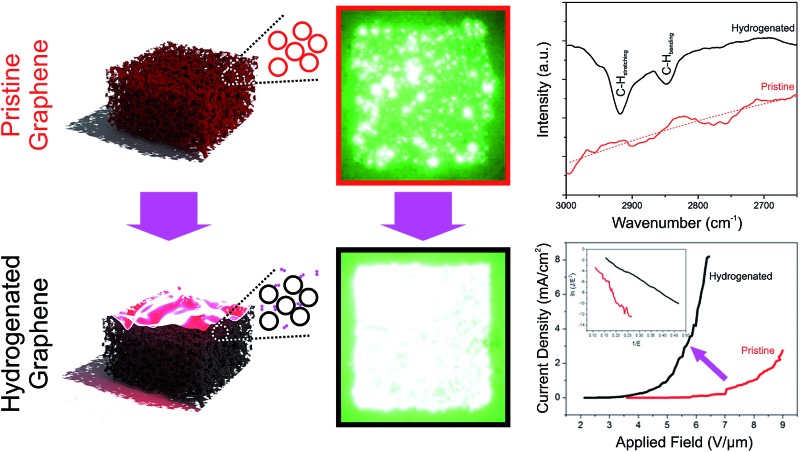
Here we report on the improved field emission performance of graphene foam (GF) following transient exposure to hydrogen plasma.

## 1. Introduction

Graphene has attracted great attention in recent years because of its outstanding opto-electronic characteristics^1–3^ and its increasingly wide range of potential applications.^4–8^ Previous studies have extensively investigated the electron emission properties of graphene sheets lying flat on substrates.^9,10^ However, little has been reported on the fabrication and performance of vertically aligned graphene on conventional substrates.^11^ Such nanoengineered structures possess unique potential in the field of vacuum nanoelectronics and, in particular, electron emission devices,^12,13^ in part, due to the ready availability of a significant number of exposed edge planes which provide a high density of efficient field emission sites.^14^ However, significant work is required to achieve practical graphene-based field emitters with low turn-on fields, high current densities, high temporal stabilities and uniform areal emission, all of which must be coupled with reliable function and inexpensive fabrication over large areas. Three-dimensional graphene foam (GF), structured graphitic meta-structures grown on nickel or copper foam templates, have recently been considered as one viable means of synthesizing these inexpensive graphene-based devices, such as super capacitors.^15–17^

The graphene sheets within GFs are seamlessly interconnected into a mechanically flexible network, endowing the material with excellent electrical and thermal conductivity, far superior to that of macroscopic, planar graphene structures derived from chemical exfoliation processes. The unique networked structure, coupled with the high specific surface area of the GF, provides outstanding electrical and morphological properties that may enable the realization of many hitherto non-manufacturable devices, such as novel field electron emission devices. However, such pristine GF is, in its as-grown pristine state, an enclosed hollow structure with few sharp edges. As such, these pristine GFs lack many suitable field emission sites and various methodologies have been investigated to improve their native emission.^18^ It has been widely reported that exposure to plasma enhances native field electron emission from graphitic allotropes.^19–23^ The varied rationale for the observed improvements have included increasing the tunneling coefficient by nanoscale tip sharpening,^19,24^ adjustment of the emitting surfaces aspect ratio and micro morphology,^25^ increasing the lattice defect density,^26^ as well as the potential removal of deleterious catalyst material in a cleaning-like process^27^ with an associated increase in the relative sp^3^ content.^28^ Amorphous, sp^2^ and sp^3^ carbon phases, along with mixtures thereof, have varied electronic characters; including their work function and electron affinity. The potential addition of dipole layers on the material surface will also adjust the interfacial tunnel barrier.

Here, we report a widely applicable, generalized post-treatment method to improve the field emission performance of GF-based electron emitters, where the as-grown graphene samples are treated with hydrogen plasma to enhance their electron emission performance *via* the derivation of a partially hydrogenated structured graphene foam. Our field emission experiments indicate that the emission efficiency can be noticeably improved following the rapid and facile plasma treatment. The possible underlying mechanism of the enhanced emission current is attributed to lattice degradation and the formation of a partially hydrogenated graphane derivative.

### 1.1 Meta-analysis

**Fig. 1:**
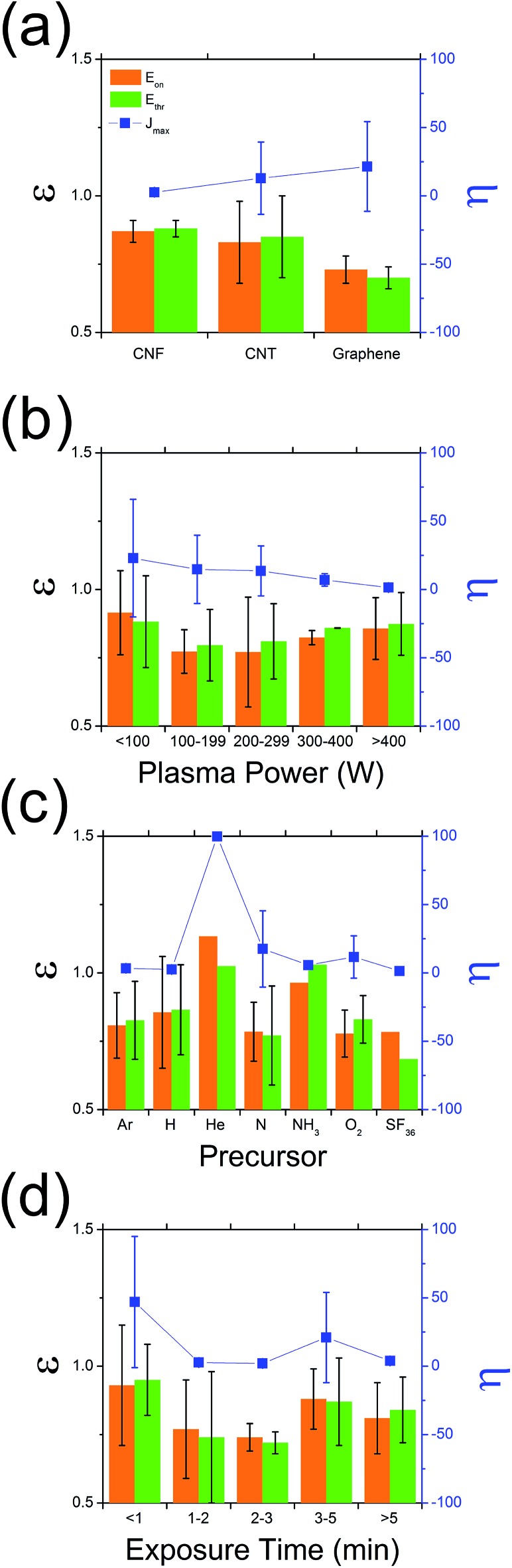
Variation in *ε* (=*E*treatedon,thr/*E*pristineon,thr) and *η* (=*J*treatedmax/*J*pristinemax) as a function of (a) graphitic substrate, (b) plasma precursor, (c) plasma power, and (d) exposure time.

A detailed meta-analysis of the literature is illustrated in Fig. 1, showing the typical variation in amplification in emission current (density), *η* = (*J*treatedmax/*J*pristinemax), and reduction in turn-on and threshold fields, *ε* = (*E*treatedon,thr/*E*pristineon,thr), for the various low dimensional graphitic allotropes (Fig. 1(a)), including graphene,^29,30^ carbon nanotubes (CNT),^19,21,22,24,27,28,31–35^ and carbon nanofibres (CNF)^36,37^ as a function of plasma precursor type, plasma power, and exposure time. Here, the subscript ‘max’ denotes the maximum measured current density, with the threshold electric field (*E*_thr_) and the turn-on electric field (*E*_on_) of the normalised current density, defined as 10% and 30% respectively. Normalization is necessitated by the intrinsic variation between studies. *η* describes the amount by which the current density improves following plasma treatment. *ε* relates to the change in shape of the diode-like current–voltage curves following plasma treatment. The emission characteristics are enhanced for *ε* < 1 and are degraded for *ε* > 1. The most beneficial plasma exposure conditions are those for which *η* → ∞ and *ε* → 0. When *ε*_on_ > *ε*_thr_, there is an increase in d*J/*d*E* at low electric fields following plasma treatment, whereas, in the case where *ε*_on_ < *ε*_thr_, there is a reduction in d*J/*d*E* associated with the plasma treatment, which manifests as a flattening of the *J*–*E* plot. In the case where *ε*_on_ = *ε*_thr_, the emission characteristics retain the same shape as the pristine samples. The mechanism which mediates such shifts is not yet entirely understood, and the underlying electron transport is currently under further investigation, to be reported elsewhere.

As evidenced in Fig. 1(a), of all the carbon allotropes reported, graphene shows the most promising enhancement following plasma treatment. For all the graphitic nanocarbons studied, plasma treatment resulted in a mean reduction of 20% in the turn-on and threshold field; though in the case for graphene we noted a mean reduction in turn-on electric field of 27% and an *η* of 29.3. The most common plasma precursor (Fig. 1(b)), Ar, showed impressive enhancement, with N showing significant promise with one of the lowest *ε* (0.78) and a simultaneously high *η* (17.0). Nevertheless, to date few studies have considered the electron emission implications of H_2_ plasma treatment, with previous data for CNTs and CNFs suggesting *ε* = 0.86. It is worth noting that H_2_ plasma perform only slightly worse than NH_3_, with the latter having a known greater propensity for the formation of atomic hydrogen required for complete hydrogenation, due to its lower thermal dissociation potential (N–H = 339 kJ mol^–1^, H–H = 436 kJ mol^–1^ (ref. 38 and 39)). No studies to date have considered the use of hydrogen plasma on super-structured graphene-based electron emitters. There is an evident stronger dependency of *η*, than *ε*, on the gas type. It is unclear as to what the underlying enhancement mechanisms are at this stage. Nevertheless, it is certainly likely that the plasma precursor will affect the resultant degree of lattice degradation and band structure of the resulting emitter. The extent to which the emitter is etched is principally dictated by the plasma power. As shown in Fig. 1(c), there is a clear trend in *η* which decreases with increasing plasma power. *ε* tends to increase with plasma power, with the exception for *P* > 100 W which we attribute to total removal of the emitter. Indeed, increasing plasma power may have a negative effect on the performance of the field emission, with <200 W performing dramatically better than for powers >200 W. However, for very low plasma powers, little to no effect was noted, with the optimal plasma conditions likely dictated by the graphitic mass of the emitter. As highlighted in Fig. 1(d), the emitters exposed for long periods of time are often totally etched, particularly for those samples consisting of a very low graphitic mass, such as monolayer graphene. These fully-etched emitters subsequently perform worse than those that had no treatment.

## 2. Graphene foam preparation

**Fig. 2:**
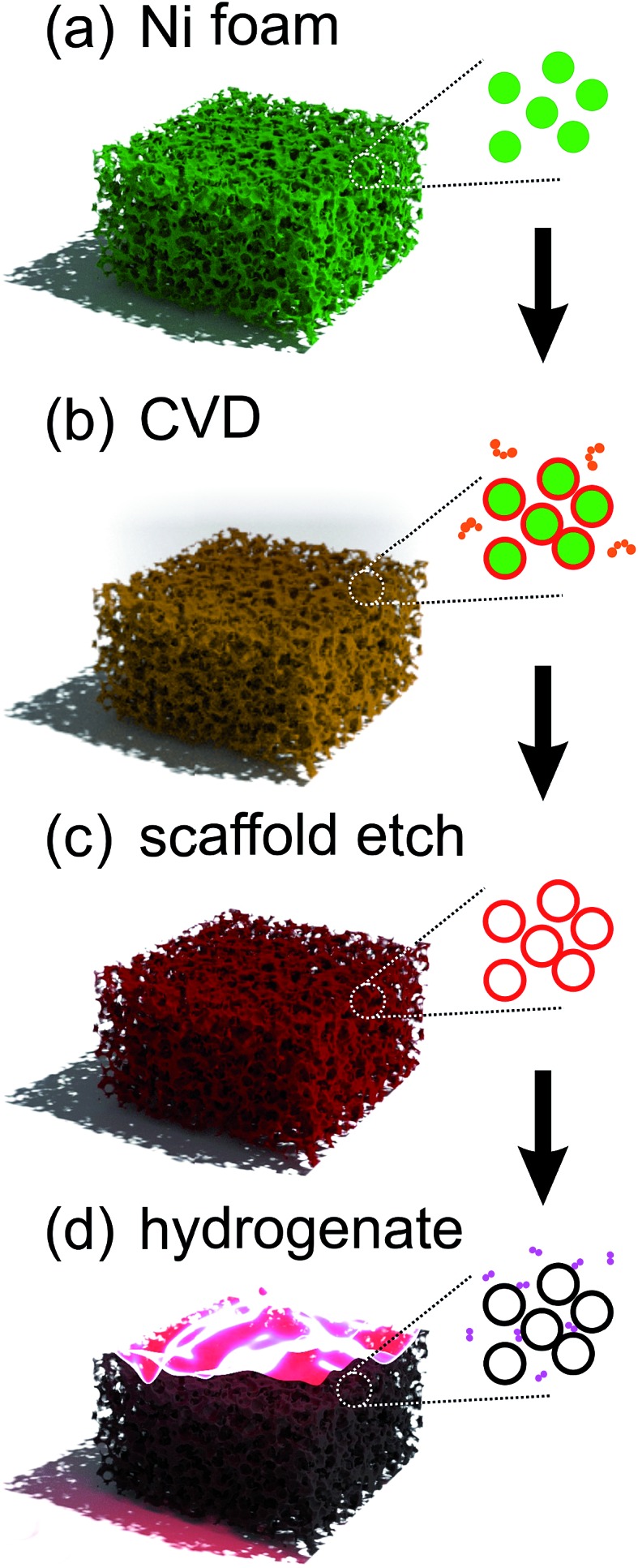
Synthesis procedure for partially hydrogenated structured graphene foam.

The detailed experimental procedure for the preparation of the GF used herein has been described in further detail elsewhere.^17^ Fig. 2 outlines the procedure. In brief, a gaseous pyrolysed carbon feedstock was introduced into Ni foam (Fig. 2(a)) by decomposing C_2_H_2_ at 900 °C, 5 mbar, resulting in the conformal growth of multi-layer (nominally trilayer) graphene around the structured metallic catalyst (Fig. 2(b)). To prevent collapse of these pristine GFs, before etching the Ni template using aqueous FeCl_3_ (Fig. 2(c)), a 100 nm support layer of poly(methyl methacrylate) (PMMA) was deposited on the GF surface. After the PMMA support layer was carefully removed, in an 80 °C acetone bath, a contiguous three-dimensional interconnected graphene monolith was obtained. Energy dispersive x-ray fluorescence (Shimadzu, EDX-8000) show residual Ni at at% with comparable trace levels to that of Fe from the etchant. No Cl peaks were noted. The GF was finally attached to a Mo substrate using carbon paste to form the field emission cathode and partially hydrogenated using a H_2_ plasma treatment (Fig. 2(d)). H_2_ plasma exposure is a common means of hydrogenation; other common approaches include liquid based classical Birch reduction,^40^ though the use of conventional PE-CVD has clear financial advantages, principle amongst which is that the same chamber can be used for the growth and hydrogenation. The pristine GF cathode structures were finally treated for 5 min in hydrogen (H_2_) plasma, at 800 W, 4 mbar, using a commercially available plasma enhanced chemical vapor deposition system (Aixtron Black Magic Pro). Plasma heating increased the sample temperature to around 300 °C. We stress here that the lengthened time and power, relative to those suggested by our earlier meta-analysis, are a direct consequence of the increased graphitic mass of the GF cathode relative to the earlier CNT, CNF and graphene materials. Moreover, the degree of plasma dissociation of the H_2_ feedstock has a known sub-linear correlation with plasma power, necessitating a higher plasma power.

Field emission properties were measured in diode configuration in a custom-built vacuum chamber with a base pressure of 5 × 10^–6^ mbar. The GF cathode was placed adjacent to an indium tin oxide coated glass anode covered with a phosphor layer, separated from the cathode assembly with two 250 μm thick alumina spacers, with a measured emission area of 1 cm^2^.

## 3. Results and discussion

**Fig. 3:**
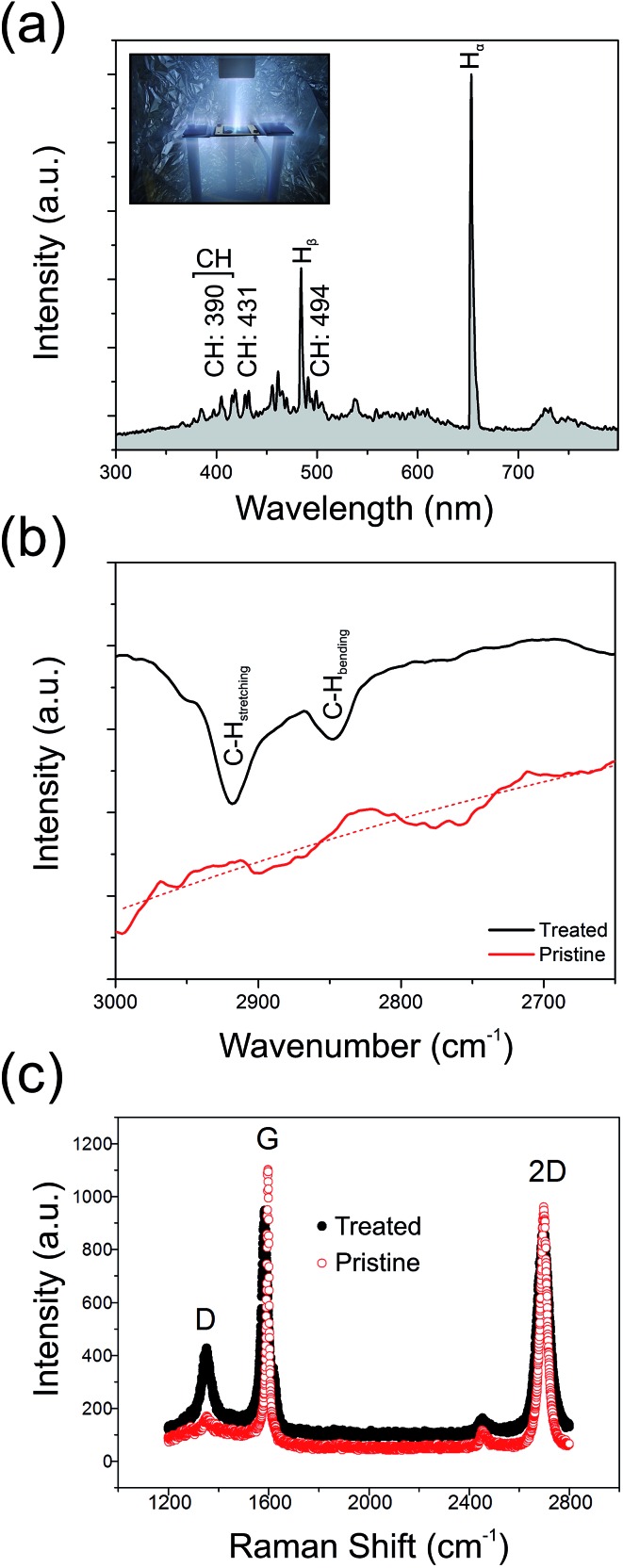
(a) An example optical emission spectrum from the H_2_ plasma during GF hydrogenation. (b) Fourier transformation infrared spectra of pristine and treated GF. (c) Raman spectra of the pristine and plasma treated GFs.

Fig. 3(a) shows the optical emission spectrum from the H_2_ plasma during hydrogenation. We note a rich spectrum containing various lines characteristic of a low carbon content hydrogen plasma. These include CH lines at 387.1 nm, 390.0 nm, 431.4 nm, and 494.1 nm, in addition to various sub-bands associated with CH(B^2^Σ^–^ → X^2^Π) emission (380–415 nm);^41^ cumulatively suggesting partial etching of the GF and liberation of atomic C into the ambient.^42,43^ Residual ion species, such as O^+^ (411.2 nm) and N^+^ (408.1 nm), are also noted. The primary H_α_ line (652.2 nm) dominates the spectrum along with several other Balmer atomic hydrogen lines. As shown in Fig. 3(b), the Fourier Transform Infra-Red Transform spectra (attenuated total reflectance FTIR; Shimadzu, IRTracer-100) of the treated samples show clear absorption peaks at 2918.2 cm^–1^ and 2851.2 cm^–1^, corresponding to the olefinic C–H stretching mode and the aromatic C–H bending mode, respectively.^44,45^ No such peaks appear in the spectrum of the pristine graphene, suggesting that plasma treatment does, at least in part, result in the formation of a partially hydrogenated graphene backbone.

**Fig. 4:**
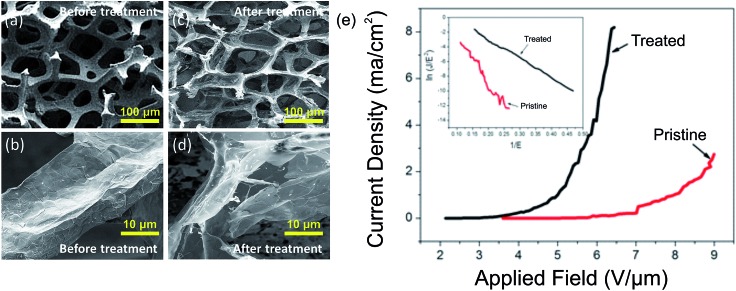
Scanning electron micrographs of pristine GF before treatment in (a) low (scale bar: 100 μm) and (b) high (scale bar: 10 μm) magnification, and the plasma treated GF in (c) low and (d) high magnification. (e) Typical variation in FE current density as a function of the applied electric field (*J*–*E*). The insert depicts the corresponding Fowler–Nordheim plot highlighting the classically quasi-metallic linear transport properties of the GF.

To better understand the underlying mechanisms for the enhanced emission, pristine and treated GFs samples were inspected using a FEI Qunata 200 scanning electron microscope (SEM) and a Horiba JobinYvon HR800 Raman spectrometer operated with a laser excitation of 532 nm and an impinging power of <5 mW. Fig. 3(c) shows typical Raman spectra for the treated and pristine GF. After the plasma processing, the intensity ratio of the defect indicative D-band (1585 cm^–1^) to the G-band (2695 cm^–1^), *I*_D_/*I*_G_, was greatly increased from 0.16 (pristine) to 0.46 (treated). Previous studies have shown that the Raman D-band primarily originates from lattice defects. Certainly, in the present case, the amount of defects within the GF have been greatly increased and may hint at one possible enhancement mechanism for the observed electron emission. The increase in graphene crystal size, *L*_a_, has been shown to be accessible through Raman spectroscopy.^46^ In the present study, the pristine GF had an *L*_a_ of 119 nm, decreasing to 41 nm following plasma treatment. This reduction by a factor of 2.8 shows an excellent correlation with the observed beneficial 2.2 factor decrease in *E*_on_, suggesting that an increase in defect areal density enhances the measured macro-scale turn-on electric field, likely due to the presence of an increased number of geometrically enhanced emission sites. Atomic hydrogen, stimulated during the hydrogen plasma treatment, is known to be readily chemisorbed onto graphitic surfaces. It has been implicated as a key mediator in lattice unzipping in graphitic carbons.^47^ Electron emission preferentially from graphene edges and small crystallites suggest that the more defective the graphene is, the higher the emission performance. However, for crystallites 1.5 nm in diameter, the work function in the pristine GF can be as high as 5.8 eV,^48^ whilst for *L*_a_ > 3 nm this value reduces to the bulk value (4.0 eV) and plateaus. In our case, our comparatively large crystallites remain unaffected by the deleterious increase in *Φ* as the GF here is not over etched. Nonetheless, the presence of a high areal density of defect sites is, broadly speaking, advantageous for enhanced field emission, in so far as the crystallites remain larger than this critical feature size. As illustrated in Fig. 4(a–d), which shows some example SEM images of the pristine and plasma treated surface morphology of the GFs, it is evident that a number of vertically aligned sharp edges were formed on the surface of GF after the plasma treatment and it is likely that the measured enhanced field emission is in part attributed to such structural augmentation, effectively providing an increased number of viable emission active sites on the surface of the GF. It is also worth noting that geometrical enhancement of the GF is implicitly associated with shifts in the bulk work function of the emitter.

Optical transmission measurements, on the broadly flat-band spectra, suggest an increase of 1.9% in the mean pore size following hydrogenation. Indeed, SEM inspection confirmed an increase in pore size, with mean pore diameters of 63.4 (±24.8) μm and 92.6 (±25.6) μm for the pristine and hydrogenated samples, respectively. Note that the suggested increase in pore size estimated from indirect optical transmission measurements are less than direct measurements by SEM, due to the structured network nature of the samples. Regardless of the exact magnitude of the pore size increase, it is likely that such increases in pore size likely manifest as an improvement in the field emission performance through reduction of nearest neighbour electrostatic shielding.

The dependence of the FE current density, *J*, on the applied electric field, *E*, of the pristine and treated chemical vapour deposited GF is shown in Fig. 4(e). The corresponding Fowler–Nordheim plots are shown in the insert of Fig. 4(e). Exposure to a cold atomic hydrogen population during H_2_ plasma treatment dramatically reduced the turn-on electric field (*E*_on_, defined as the macroscopic electric field to produce a current density of 10 μA cm^–2^); the nominal *E*_on_ reduced from 5.6 V μm^–1^ to 2.5 V μm^–1^. A lowering of the threshold field (*E*_th_, defined as the field required to produce a current density of 1 mA cm^–2^) was also noted, and was reduced from 8.1 V μm^–1^ to 5.0 V μm^–1^, values consistent with those reported elsewhere for other graphitic nanocarbon allotropes.^49^ Both the pristine and treated FE spectra exhibit near-linear behavior in the measurement range considered, which can be attributed to the quasi-metallic transport character of the emitter. The emission current–voltage characteristics have been analyzed by Fowler–Nordheim theory, of the form;
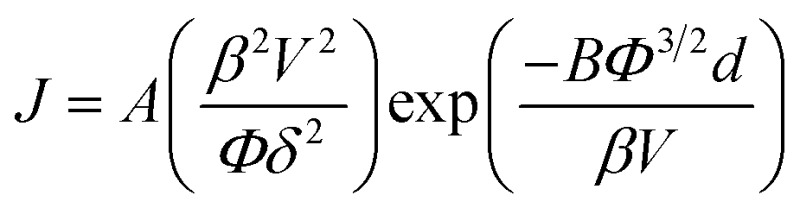
where *J* denotes the current density, *A* = 1.56 × 10^–6^ (A V^–2^ eV), *B* = 6.83 × 10^9^ (V eV^–3/2^ V m^–1^), *Φ* is the emitter work function, *E* is the macroscopic applied electric field, *d* is the distance between the anode and the cathode, and *V* is the applied voltage. Here, the *β* represents a matrix dependent field enhancement rather than a conventional single emitter based aspect-ratio-dependent metric.

Assuming *Φ* is 5.0 eV for graphitic materials,^50^ the mean field enhancement factors of treated GF and pristine GF were calculated as 3400 and 1100, respectively, suggesting a distinct increase in the average whisker-like features within the GF following plasma treatment. Even in the likely case that the treated GFs have a shifted *Φ*, to which we will return to discuss later, the field enhancement factors still remain significantly larger than those of the pristine samples as the *Φ* shifts are arithmetically minor. During hydrogen ion bombardment, much residual *a*-C (amorphous carbon) is removed, along with other non-graphitic organics. Alongside this there is general lattice etching and hydrogenation, the latter of which was proposed elsewhere in the case of carbon nanotubes.^28^ This etching process generates a large number of the defects and sharp edges on the surface of the GF, hence modifying the local electric field, as previously evidenced.^51^

Though an increasing number of readily emitting edges are likely formed following plasma exposure, there are benefits associated with using hydrogen over other plasma precursor species. Shifts in the surface *Φ* are known to dramatically bolster the FE performance of nanocarbon emitters.^52–56^ Using a similar PE-CVD approach, Baldwin *et al.* ^57^ reported an *I*_D_/*I*_G_ ratio of ∼2, resulting in graphene with an *L*_a_ of 8 nm. Increasing the defect and dangling bond density is likely to increase the propensity towards hydrogenation with notable increases in the number of terminated C–H bonds. Typically, observed reductions in *L*_a_ are due to hydrogenation, and possible graphane production, principally at domain boundaries. Baldwin *et al.* suggested a hydrogen content of <10%,^57^ most of which is likely localised to the inter-granular defect zones. Under our optimized conditions, our Raman spectra suggest a partial hydrogenation, and thus areal graphane content, of approximately 3%. Though low, this nevertheless suggests a potential decrease in the mean emitter surface work function of <0.1 eV,^48^ which, when considered in the context of a quasi-metallic emitter with well-fitted Fowler–Nordheim tunneling, is sufficient to increase the beam current by around 30% at a given bias.

In the case of H_2_ plasma treated carbon nanotubes, Zhi *et al.* showed a reduction in the turn-on field from 3.9 to 2.9 V μm^–1^,^51^ whilst for Ar ion irradiation, Kim *et al.* and Qi *et al.* evidenced reductions from 5.5 to 2.0 V μm^–1^ and 3.9 to 2.2 V μm^–1^, respectively.^23,58^ It has been suggested that a surface C_δ–_–H_δ+_ dipole, which may reduce the electron affinity, following H_2_ plasma treatment, along with the formation of a high density of lattice defects; both of which enhance the samples propensity to emit. It is also likely that the emission is further enhanced due to the removal of preferentially etched catalyst particles and the formation of extremely high aspect-ratio sub-nano tips, which may very well increase the local electronic field further.

An increased number of localized defect states near or above the Fermi level enhance the emission given the higher tunneling probability, with the potential for inter-granular *a*-C and graphitic phases further enhancing the emission. These reactive defect sites readily emit, but also readily bind to various gaseous species in the ambient. It is this edge passivation which is central to the observed emission enhancement; hydrogenated edges present a low barrier of 4.1 eV, whilst this is increased to 4.6 for O_2_ passivated edges. Indeed, hydrogen passivation has been shown elsewhere to reduce *Φ* of graphitic carbons to as low as 3.98 eV, a reduction of around 0.5 eV,^48^ which has the theoretical potential to increase the field emission current by between one and two orders of magnitude,^59^ consistent with our earlier empirical findings where we noted a 40-times increase in the maximum measured emission current density, *J*_max_. Indeed, fully H-saturated (111) diamond surfaces have shown to reduce the *Φ* of the emitting surface by up to 0.4 eV.^60^

Direct exposure to an electron beam following exposure to ammonia vapour has also been shown to result in the formation of partially hydrogenated graphene, a consequence of the dissociation of absorbed H_2_O and NH_3_ sourced H^+^ ions and hydrogen radicals.^61^ Indeed, it is likely that during electron emission chemisorbed H_2_O will dissociate and hydrogenate the graphene substrate. A ballasted-like emitter response will then be elicited, with these increasingly resistive hydrogenated zones controllably limiting the total current from the dominating tips, allowing morphologically less-favorable tips to engage, thereby increasing the total emission current and emission uniformity.

Graphene hydrogenation is reversible.^62^ Heating hydrogenated graphene to temperatures in the order of 600 °C induces near complete dehydrogenation.^63^ Such dehydrogenation would likely revert, in part, the emission enhancements observed here, particularly those associated with the adjusted surface *Φ*. Significant heating is not uncommon during field emission measurements,^64,65^ however, notwithstanding, this local hydrogenation *via* the electron beam assisted dissociation of chemisorbed H_2_O may largely counter-act the unavoidable thermally stimulated dehydrogenation of the graphene substrate. Nevertheless, such electron beam stimulated maintenance of the hydrogenation is certainly transient, and under maintained high-vacuum conditions will rapidly be exhausted, compared to the typically year-long DC life-time of most field emission sources.

**Fig. 5:**
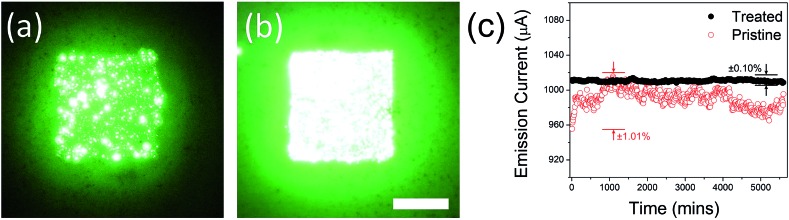
Emitter temporal and spatial uniformity. Example integrated intensity emission images of (a) pristine GF and (b) plasma treated GF cathodes (scale bar: 5 mm). (c) Typical temporal stability profiles of the pristine and treated GFs.

Integrated emission images of the pristine GF and treated GF cathodes are shown in Fig. 5(a) and (b), respectively. All images were acquired at an emission current of 0.5 mA. Before plasma treatment, the image uniformity was very poor with a significant number of hot spots. Along with a near doubling of the apparent brightness, following plasma treatment the GF cathode showed a notable increase in emission uniformity; the pristine GF had a 38.8% variation (1*σ*) in emission uniformity, whilst following plasma treatment the GF showed only a 10.7% variation. It is likely that the plasma exposure increased the macro and microscopic uniformity of the emitter, preferentially etching those tips which would have otherwise dominated the emission. Such improvement in the spatial uniformity are similarly coupled to improved temporal stabilities. Fig. 5(c) shows typical temporal stability profiles of the pristine and the treated GFs, measured at biases of 8 V μm^–1^ and 5 V μm^–1^, respectively, in order to ensure the emission of equivalent currents. This 60% larger driving field is necessary to stimulate an equivalent emission current that has clear practical ramifications. We note that the treated GF shows a significantly reduced temporal variation of only ±0.10%, compared to the pristine GF (±1.01%). As we have previously reported,^66^ the pristine GF already offers somewhat impressive temporal stability, though our evidence suggests that plasma treatment of these already stable emitters further enhances their temporal stability by around an order of magnitude. Hydrogenation has also been shown to increase the electrical resistance of bulk graphitic superstructures such as these,^67^ transfering their transport characteristics from those of a semi-metal to those increasingly being semi-conducting.^62^ This shift functionally manifests as an emission ballasting element, further preventing over emission from the dominate sites. Nevertheless, it is also possible that the plasma treatment may increase the bulk resistivity of the emitter. As previously alluded to, this may be an entirely deleterious outcome. Indeed, such modest increases, say a few percent, will likely function as a ballast resistance. Indeed, like many other research groups, we have previously studied the merits of integrated serial ballast resistance in their field emitters in order to current-limit the resistance.^68–70^ Modestly increasing the effective bulk resistance of the treated GF foam relative to the pristine samples may in fact underpin the enhanced temporal stability observed. The bulk resistivity of the treated GF foam was 22.5 ± 3.8 Ωcm, only a few percent higher than the untreated sample. Plasma treatment did not significantly alter the bulk conductivity of the GF, which was suggested during SEM inspection given the consistent grey scales between images.

## 4. Conclusions

Here, the field emission behavior of multi-layer graphene foams treated by hydrogen plasma have been investigated and used to realise the first graphene–graphane hybrid electron emitter. The fabricated hydrogenated graphene emitters demonstrated greatly improved electron emission performance following hydrogen plasma treatment, with the graphene–graphane hybrids showing a 44% reduction in turn-on field, a 394% increase in maximum emission current, and a four-times improvement in emission uniformity. We rationalise the observed enhancement in the emission performance by the evolution of lattice defects and partial hydrogenation of the graphene substrate. This increases the geometrical enhancement factor of the graphitic superstructure whilst simultaneously augmenting the mean surface work function. We have shown that of the available plasma precursor gases, hydrogen may be one of the more effective in deriving a controlled etching and surface work function adjustment atmosphere. These results indicate that plasma treatment is an effective and widely applicable method to improve the field emission properties of many graphene-based field emission cathodes, with graphane emitters in particular being one such promising candidate material for future nanoengineered electron guns.

## Note added after first publication

This article replaces the version published on 14th December 2015, which contained an error in the title.
